# Status of surveillance and routine immunization performances in Amhara Region, Ethiopia: findings from in-depth peer review

**DOI:** 10.11604/pamj.supp.2017.27.2.10755

**Published:** 2017-06-09

**Authors:** Gebre Asmamaw Lakew, Eshetu Wassie, Ayesheshem Ademe, Ayalneh Fenta, Solomon Wube, Mihret Werede, Achenef Kidane, Leulseged Mekonnen, Teklehaimanot Gebre Hiwot, Kathleen Gallagher

**Affiliations:** 1 World Health Organization, Ethiopia; 2 Amhara National Regional States, Health Bureau, Ethiopia

**Keywords:** Peer review, surveillance, AFP, measles, Non polio AFP rate, stool adequacy rate, case based, immunization

## Abstract

**Introduction:**

Trend analyses of non-polio AFP and stool adequacy rates in Amhara Region showed optimal performance over the years. However, sub regional gaps continue to persist in certain zones where the reasons for low performance were not well documented. The objective of this study was to assess the performance of the disease surveillance and immunization system in Amhara Region, Ethiopia with emphasis on low performing woredas and zones.

**Methods:**

A descriptive cross-sectional study was conducted from July 2-10, 2015 to assess the structure, core and support surveillance functions in five zones and two town administrations that were purposively sampled based on differing performances, geographic location, and history of vaccine preventable disease outbreaks among others.

**Results:**

Of the 82 sites reviewed, 71 (87%) have a designated surveillance focal person. Less than half 36(44%) of these focal persons have written terms of reference. Twenty-six (93%) of the health offices had a written surveillance work plan for the fiscal year. Only 17 (81%) of woreda health offices and town administrations had prioritized active surveillance sites into high, medium and low during the last 12 months. Only 4(17%) had independent active case search visits to these sites as per the priority. Seventy-eight (95%) and seventy-seven (94%) sites have a designated immunization focal person and updated EPI performance monitoring charts, respectively. There had been vaccine stock out in the 3 months before assessment in 28 (34%) of the sites.

**Conclusion:**

Though there is an existence of well-organized surveillance network with adoption of the integrated disease surveillance and response, gaps exist in following the standard guidelines and operation procedures. Improvements needed in reporting site priority setting and regular visiting for active case search, outbreak investigation and management, vaccine supply and overall documentations.

## Introduction

Effective communicable diseases control relies on effective surveillance and response systems that promote coordination and integration of surveillance functions. Recognizing this, an initiative to strengthen the disease surveillance system by promoting integration of surveillance activities was started in 1996 in Ethiopia. Later in 1998 the World Health Organization Regional office for Africa (WHO-AFRO), following the resolution of the 48th assembly, started promoting Integrated Disease Surveillance and Response (IDSR) for all Members States to adopt as the main strategy to strengthen national disease surveillance systems [1]. Ethiopia as a Member State adopted the IDSR strategy, which is district centered and outcome-oriented. Based on the steps recommended by the strategy, the Federal Ministry of Health (FMOH) of Ethiopia and its development partners conducted an assessment of the country's surveillance systems in October 1999 and subsequently prepared a 5-year national implementation plan [2]. Vaccine Preventable Disease (VPD) surveillance, including acute flaccid paralysis (AFP) and measles surveillances, is one of the operational components of IDSR. In 2009, FMOH restructured its health programs and designed a more comprehensive approach for public health surveillance and responses including recovery from public health events named Public Health Emergency Management (PHEM). IDSR and VPD surveillance were included in PHEM. Public Health Emergency Management (PHEM) was designed to be implemented directly by the Regions [2]. Amhara, one of the nine Regions and two City Administrations of Ethiopia, implements VPD surveillance within the framework of the IDSR strategy as part of PHEM. We describe the process and the result of Amhara Regional State peer exchange integrated disease surveillance review which was conducted from 2 to 10 July 2015. The main objective was to assess the performances of disease surveillance and routine immunization in Amhara Region with particular focus on VPD surveillance and the extent of its integration into PHEM and thereby share experiences among WHO officers and their government counterparts.

## Methods

**Study area:** Amhara Region is one of the nine administrative regions in the country. It is the second most populated region in Ethiopia with a projected total population of 20,398,999 (from 2007 Census) as per the national Central Statics Authority (CSA) with a mean annual growth rate of 1.8% [3]. The Region shares boundaries with four Regional States of the country (Oromyia, Tigray, Afar and Benishangul-Gumuz) and The Sudan. The Region has 10 zones and three town administrations which are further divided into 167 woredas (similar to districts) which further comprise a total of about 3,431 kebeles (sub-districts), of which 318 are urban.

**Study design:** we conducted a cross-sectional study between 2-10 July 2015 in South Gondar, North Gondar, West Gojjam, South Wollo zones and two town administrations (Bahirdar and Dessie).

**Sampling:** five zones and two town administrations were purposively sampled and visited for the review based on recent surveillance performances and risk of wild or vaccine derived polio virus importations .We included those areas owing to the sub-optimal AFP surveillance performances during the two years prior to the review and zone bordering Sudan with history of imported circulation of wild polio virus in the past. Zone and woreda health offices, health facilities, and a holy water site were selected for review. Three to five woreda health offices and two to three health facilities in each of the woredas were included for the review in most of the areas. One holy water site was also visited. We tried to include both good and poor performing woredas in the selected zones. Health facility selection considered representation of all types of facilities in the level of care and geography (urban-rural).

**Measurements/variables:** the following variables were considered for both health offices and health facilities: availability of focal persons and their training status in the last two years, terms of reference for surveillance and availability of formats and guidelines, supervisory support from higher levels, and documentation status of cases and outbreaks. The following variables were considered for woreda and zonal health offices: availability of surveillance work plan, written supervision plan, supervisory checklist that adequately addresses surveillance, records for active surveillance visits, review meetings on surveillance, availability of coordinating committees and rapid response teams, availability of line lists of reported AFP, measles and neonatal tetanus cases, and monitoring of selected AFP and measles surveillance indicators. Prioritization of sites and written schedule for active surveillance were assessed for woredas and town administrations only. Clinician sensitization during last 6 months and availability of standard case definitions or reminders were variables considered for only health facilities.

**Data collection:** the primary tool for data collection was a structured questionnaire adapted from the standard WHO - AFRO tool for external surveillance review. The tool consisted of two sets of questions: the first set can only be administered to zonal and woreda health offices and the second set only apply to health facilities. Generally, the questionnaire was designed to assess the organization of surveillance at each level, active surveillance system, case-based surveillance performance and documentations [4]. The tool also assessed PHEM implementation and its monitoring, outbreak detection, response and documentation, and the effects of the polio eradication initiative (PEI) resources on general surveillance and other programs, and key aspects of immunization. The review teams consisted of six WHO surveillance officers and six government surveillance counterparts who received orientations on the data collection tools and procedures. One WHO surveillance officer and a government counterpart who is from the zone to be reviewed, consists of one review team and thus, expected to review all the selected sites in one zone. Each WHO surveillance officer was assigned to a different zone to independently assess his peer's zone and also share experiences of surveillance in the zone. The tool was administered through interviews of surveillance focal persons or their subordinates, heads of health offices, health facilities and holy water site. The team also conducted reviews of surveillance related documents (e.g., meeting minutes, surveillance plans, supervisory checklists and active surveillance sites prioritization documents) at the various levels. Observations of key service delivery practices, and discussions with heads and senior members of the visited sites were made to get their general views of surveillance system and their recommendations for further strengthening of WHO's support.

**Data analysis:** primary and secondary data were entered, cleaned, and analyzed in MicrosoftTM Excel 2010. Proportions were calculated for selected variables and key indicators.

## Results

Peer WHO Medical Surveillance Officers and regional and zonal PHEM officers reviewed a total of 82 reporting units consisting of five zones, two town administrations, 21 woredas, and 54 health facilities. One holy water was visited but excluded from the analyses because of incomplete information.

### Organization status of surveillance

Written surveillance work plan was available for 26 (93%) of the 28 zonal and woreda health offices reviewed. There was a written supervision plan and supervisory checklist that covers surveillance in 23 (82%) and 26(93%) of health offices visited, respectively. Of the reviewed offices and health facilities, 71 (87%) had a designated surveillance focal person. About three-fourth (76%) of these surveillance focal persons had a refresher training in the last two years. Only 36(44%) of the offices and health facilities have written terms of reference for surveillance. Out of the reviewed health offices and facilities, 71 (87%) have operational surveillance guidelines. Standard case definitions on AFP, measles and NNT were available and posted in outpatient departments of 50 (93%) of the 54 health facilities visited (Table 1).

**Table 1:** Organization of disease surveillance in Amhara Region, July 2015

Level/Variables	Number	Yes
**Woreda and zonal health offices only (n=28)**		
Availability of surveillance work plan	28	26(93%)
Availability of written supervision plan that covers surveillance	28	23(82%)
Availability of supervisory checklist that covers surveillance	28	26(93%)
**Health offices and health facilities (n=82)**		
Availability surveillance focal person	82	71(87%)
Focal persons received refresher training last 2 years	71	54(76%)
Availability of surveillance terms of reference	82	36(44%)
Availability of surveillance guide lines	82	71(87%)
**Health facilities only (n=54)**		
Availability of standard case definitions	54	50 (93%)

### Surveillance network and active surveillance sites

Availability of prioritization of reporting sites was assessed in 21 woreda and two town administration levels and it was found that only 17 (81%) of them had potential reporting sites prioritized in to high, medium and low during the last one year. Private health facilities, holy water sites and traditional practitioners included in the prioritization of surveillance units by 21 (70%) of woreda and town health offices visited. Only nine (39%) of woredas and town administrations have written schedule for visiting priority reporting sites. Only four (17%) of woredas and town administration health offices have made regular active surveillance visits based on their plan during the last 6 months. There was a written evidence of regular active case search within health facilities (e.g. in the form of weekly register review and signature by facility focal person) in more than two thirds (70%) of health facilities visited. Almost all (96%) of 23 woreda and town administration health offices had made one or more independent active surveillance visits to reporting sites during the six months prior to the visit. But, only 15 (65%) had documented records (e.g. filled management tool or integrated checklist) for the active case search visits to these reporting sites. Both health offices and health facilities were inquired if a supervisor from higher level visited them for surveillance, and it was found that 75 (91%) of the sites were visited during the past 6 months. The supervisory visit was followed by written feedback in 58 (78%) of supervised sites. About two-thirds (63%) of visited health facilities have received clinician sensitizations on surveillance during six months prior to the assessment. Surveillance review and/or monitoring meetings mostly integrated with review of other health programs were conducted in all of the 28 health offices and town administrations during the past 12 month. Of the health offices and health facilities with laboratory services, 55 (72%) of them had laboratory staff actively involved in surveillance activities. Almost all (96%) of health offices have coordinating committees for surveillance activities either in the form of health emergency taskforce or rapid response team (Table 2).

**Table 2:** Surveillance network and active surveillance sites in Amhara Region, July 2015

Variables	Number	Yes
Have reporting facilities been prioritized into high, medium and low priority	21	17(81%)
Are there any other key community informants included in active surveillance	82	35(43%)
Knowledge of health workers in case definition of priority diseases	82	78(95%)
Prioritization of reporting units updated	30	16 (53%)
Are private health facilities included in active surveillance activities	30	21(70%)
Has a supervisor visited this site for surveillance activities in the last six months?	82	75(91%)
Are there any supervisory reports/feedbacks on surveillance available?	74	58(78%)
Are laboratories involved in surveillance activities (e.g., as member of RRT, collect and process samples, assist weekly report compilation) at this level?	79	53(67%)
Are there any coordinating structures for surveillance (PHEM/emergency task force?)	81	62(77%)
Was surveillance monitoring/review meeting conducted in the last 12 months?	81	72(89%)

### Case based surveillance

**Figure 1:**
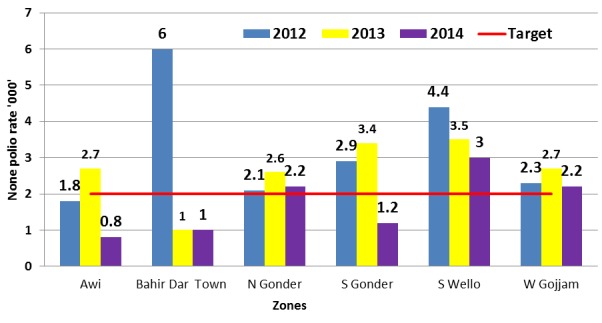
Distribution of the non- polio AFP rate by Zone, Amhara Region, Ethiopia, 2012-2014

**Figure 2:**
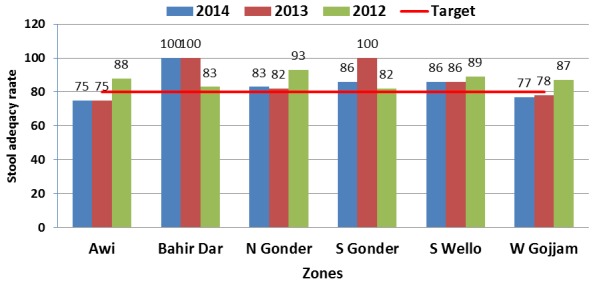
Distribution of stool adequacy rate by zone, 2012 -2014 Amhara region, Ethiopia

Three years (2012-2014) trend analyses of AFP detection rates for the zones reviewed showed that Awi and Bahir Dar had non-polio AFP rates below 2 per 100,000 under-15 years of age population for two of the three years reviewed. Detection was also low (1.2/100,000) in 2014 for South Gondar. Stool adequacy rate was below the target 80% during two of the three years in both Awi and West Gojjam zones (Figure 1, Figure 2). Majority ,75 (91%) ,of the health workers interviewed in all of the 82 sites have knowledge of standard case definitions and case investigation procedures including sample collection for AFP and measles cases (Table 3). Of the 71 sites that have reported at least one AFP case during the last three years, only 41 (58%) of them had documented copies of those reported cases. Of the 77 sites that reported at least one measles case, only 47 (61%) have copies of case investigation forms for the reported cases during the last three years. Sixty-two (76%) sites said to have reported one or more neonatal tetanus cases but only 16 (26%) of them had copies of the reported cases during the three year period. AFP and measles surveillance indicators were regularly calculated and monitored in 12 (43%) and 13 (46%) of health offices, respectively. Readiness for AFP and measles case investigations was also reviewed. More than two-thirds (68%) and about a third (36%) of the 76 offices and health facilities other than health posts have blank AFP investigation form and standard stool collection cup, respectively. Blank measles case investigation forms were available in only 45 (59%) of similar sites reviewed (Table 3).

**Table 3:** Status of key activities of case-based AFP, measles and neonatal tetanus surveillance, Amhara, Jul 2015

	Number	Yes	%
Knows case definitions and sample collection procedure	82	75	91
Have AFP Case file	71	41	58
Have measles case file	77	47	61
Have neonatal tetanus case file	62	16	26
AFP surveillance indicators monitored	28	12	43
Measles surveillance indicators monitored	28	13	46
Have blank AFP report form	76	52	68
Have standard stool cup?	76	27	36
Have blank measles report form	76	45	59

### Public Health Emergency Management Performances

PHEM activities are being implemented at all, 82 (100%), sites reviewed. Of all sites visited, 64(78%) have integrated case definitions of IDSR priority diseases. The national PHEM guidelines were available in 69 (84%) of the sites. A line graph was available and used for trend analysis in 55 (67%) of the 82 sites (Table 4). Of the 66 suspected diseases outbreaks reported from January to June 2015, only 45 (68%) were investigated and 23 (51%) of these investigations were done properly as per national guidelines. The proportion of laboratory confirmation of outbreaks (all of them measles) was 47 (71%) and there was proper response in 33 (50%) of the 66 reported outbreaks (Table 4).

**Table 4:** Public Health Emergency Management (PHEM) performance status, Amhara Region, Jul 2015

Key PHEM issue	Number	Yes	%
PHEM implementation started	82	82	100
Availability of National PHEM guideline	82	69	84
Availability of PHEM report format	82	78	95
IDSR diseases' definitions posted?	82	64	78
PHEM trained health worker available?	82	69	84
Line graph being used for trend monitoring?	82	55	67
Rumor log book used (health centers, health offices)?	71	52	73
No. of investigated outbreaks	66	45	68
No. properly investigated	45	23	51
No. of lab confirmed outbreaks	66	47	71
No. of outbreaks with proper response	66	33	50
Polio resources supported other programs?	28	28	100

### Routine immunization

A designated Expanded Program of Immunization (EPI) focal person was available in 78 (95%) of sites and there was a trained cold chain technician in 37(48%). EPI monitoring tools (i.e., tally sheets and reporting formats) were available in 93% and EPI coverage performance monitored on a monthly basis in 77 (94%) of sites. There had been vaccine stock out in the 3 months before assessment in 28 (34%) of sites (Table 5).

**Table 5:** Assessments result of routine immunization performance Amhara Region, July 2015

Variable	Number	Yes
Designated EPI focal person available	82	78 (95%)
EPI micro plan available	82	78 (95%)
Vaccine stock out the last three month	82	28 (34%)
Availability of trained cold chain technician	77	37 (48%)
Cold storage space adequate for this level?	80	71 (89%)
Availability EPI monitoring tools	55	51 (93%)
EPI coverage performance monitored monthly bases	82	77 (94%)

## Discussion

This study addresses an important peer exchange surveillance and immunization review in selected zones and woredas of Amhara regional state. The main finding from this evaluation was that there were still some challenges to the functioning of the disease surveillance and immunization systems in some of the visited woredas [5–7]

In the majority of reviewed woredas and health facilities, we found that 71 (87%) visited sites have designated surveillance focal persons. However, less than half 36 (44%) of the health offices and health facilities have clearly written and communicated surveillance terms of reference and standard operating procedures for their focal persons. Though not well-structured and not detailed with measurable indicators, almost all, 26 (93%), of the health offices reviewed have written surveillance work-plans, mostly as a section of annual integrated health plan.

Though officers were aware of the number, type and location of their reporting sites, not all surveillance focal persons have exhaustively listed reporting sites organized into high, medium, and low priorities. Conducting active case search by surveillance focal persons is one of the major activities for diseases targeted for eradication and elimination but this activity hardily seen in the reviewed sites. Woreda surveillance focal persons were key persons to ensuring of active surveillance in the reporting sites. Except irregular integrated supportive supervisions, these personnel were not performing independent active case search on regular basis due to financial limitations and multiple responsibilities they had. The results of surveillance performance analysis were not being used at all levels to stimulate activities to improve surveillance performance [8]. All levels knew the correct case definitions of AFP, measles, and to a lesser extent neonatal tetanus. The respondents at all levels demonstrated good knowledge of correct procedures for investigating AFP and measles cases. However gaps in knowledge of case definitions, national procedures for outbreak investigation and response do exist especially at health facilities. Except in few woredas, satisfactory mechanisms had been put in place to ensure case investigation forms and stool kits are available for investigation of AFP cases [9]. Documentation of the activities carried out including case files of reported cases of reportable diseases were very low and also lacking completeness.

We have found that there was measles and other priority diseases outbreaks in the visited areas but not all outbreaks were laboratory confirmed. The involvement of laboratories in surveillance activities (e.g., as member of rapid response team, collect and process samples, assist weekly report compilation) at all levels is very limited (8). The biggest concern we found from our review was that not all outbreaks specifically measles were properly investigated, lab confirmed and appropriately responded [10]. The major reason given was that there was no adequate vaccine stock for outbreak response immunization. Almost all immunization program implementing sites were found to have relatively better system performances in terms of planning, implementing and monitoring. However, high rate of vaccine stock outs and insufficient numbers of cold chain technicians are the major weaknesses for proper functioning of the program.

Finally, we found that polio related resources were used to strengthen other health programs particularly through integrated trainings, review meetings and supervisions, technical supports from polio funded WHO staff, and budgetary supports for other PHEM and routine immunization activities,

**Limitations of the study:** the main limitations include the selection of poor performing zones, inclusion of small number of woredas and health facilities due to financial and time constraint. Thus, the current findings cannot be generalized for the whole region. Despite these limitations, however, the study revealed challenges and successes of disease surveillance in the study areas with viable recommendations. Moreover, experiences shared among World Health Organization surveillance officers and their government counter parts. This exercise enabled WHO medical surveillance officers as well as the government surveillance focal person to independently identify strength and weakness of the system.

## Conclusion

In conclusion, disease surveillance system is better organized, planned, implemented and monitored at all levels of Amhara region. However, there are major gaps in following the nationally adopted standards and guidelines both in routine surveillance and outbreak managements. Immunization program is constrained by high rates of vaccine stock outs and inadequate numbers of cold chain technicians. Furthermore, particular gaps exist in Awi, Bahir Dar Town and South Gondar Zones of the region. Hence, woreda and health facility focal persons should properly formulate operational plans for strengthening active case search with realistic prioritization of reporting sites to be visited; the planned schedules should be strictly adhered to. The regional or zonal or Woreda level should analyze data and closely monitor surveillance performance indicators, properly investigate and respond to outbreaks. Overall surveillance documentations should be improved. A system should be in place to ensure sustainable supply of vaccines for the region.

### What is known about this topic

- In 1998 the World Health Organization Regional office for Africa (WHO-AFRO), following the resolution of the 48th assembly, started promoting Integrated;
- Disease Surveillance and Response (IDSR) for all Members States to adopt as the main strategy to strengthen national disease surveillance systems;
- Ethiopia as a Member State adopted the IDSR strategy, which is district centered and outcome-oriented.

### What this study adds

- It builds the capacity of zonal and woreda (district) Government focal person who participated in the assessment;
- It will also be an input for surveillance planning for the study areas.

## Competing interests

The authors declare no competing interests. The views expressed in the perspective articles are those of the authors alone and do not necessarily represent the views, decisions or policies of the institutions with which they are affiliated and the position of World Health Organization.
